# Spanish Murders and Brigandage

**Published:** 1881-07

**Authors:** 


### SPANISH MURDERS AND BRIGANDAGE.

IN Spain there is not much actual murder, but there is rampant brigandage, which only stops short of murder providing it can rob without it. Even in Madrid itself, in one of the finest and most frequented streets, a member of the Senate was, only two years ago, kept prisoner in his own bedroom and threatened with death until he paid the ransom demanded of him.

Bands of robbers, as is only too well known, haunt the mountain districts even in the neighborhood of the capital. The brigands are said to have friends in very high places; they exercise a terror which prevents quiet people from daring to give evidence against them; they walk out of prison if they are put into it, and when they hold land they pay to the Government just the amount of taxes that they think convenient.

Justice, again, is slow in most countries, but in Spain it scarcely moves at all. Every process is secret, and everything is carried on in writing. The pile of papers heaped up in reference to the murder of Gen. Prim ten years ago mounts up and up; but it is not even yet thought high enough, and a trial seems as far off as ever. The Government is as unable as any one else to insure a speedy conviction and if it really wants to get rid of notorious criminals, it shoots them on the pretext that they are trying to escape.

### A QUEEP WAY OF BUILDING HOUSES.

There are in the world many queer ways of making houses, and one of the queerest is found in the City of Palembang, in Sumatra. The town extends for three or four miles on both sides of a rather wide river, and both shores are lined with houses. First comes a row built upon piles which are driven into the bottom of the river, and outside of that another row resting on great bamboo rafts, which are held by cables of rattan to the piles of the next houses. Of course these rafts rise and fall with the tide, and the doars open upon the water, so that they are reached by boats. One can buy anything there is for sale in this town without getting out of his boat. The people are Malays, and it is said that they never build a house on dry land if they can find water to set it in, and never go anywhere on foot if they can reach the place in a boat.

